# Chernobyl seed project. Advances in the identification of differentially abundant proteins in a radio-contaminated environment

**DOI:** 10.3389/fpls.2015.00493

**Published:** 2015-07-06

**Authors:** Namik M. Rashydov, Martin Hajduch

**Affiliations:** ^1^Department of Biophysics and Radiobiology, Institute of Cell Biology and Genetic Engineering, National Academy of Sciences of Ukraine, KievUkraine; ^2^Department of Developmental and Reproduction Biology, Institute of Plant Genetics and Biotechnology, Slovak Academy of Sciences, NitraSlovakia

**Keywords:** soybean, flax, ionizing radiation, ecology, experimental design, seed filling, 2-DE, mass spectrometry

## Abstract

Plants have the ability to grow and successfully reproduce in radio-contaminated environments, which has been highlighted by nuclear accidents at Chernobyl (1986) and Fukushima (2011). The main aim of this article is to summarize the advances of the Chernobyl seed project which has the purpose to provide proteomic characterization of plants grown in the Chernobyl area. We present a summary of comparative proteomic studies on soybean and flax seeds harvested from radio-contaminated Chernobyl areas during two successive generations. Using experimental design developed for radio-contaminated areas, altered abundances of glycine betaine, seed storage proteins, and proteins associated with carbon assimilation into fatty acids were detected. Similar studies in Fukushima radio-contaminated areas might complement these data. The results from these Chernobyl experiments can be viewed in a user-friendly format at a dedicated web-based database freely available at http://www.chernobylproteomics.sav.sk.

## Introduction

Radioactive minerals have accumulated on the Earth’s surface since early Achaean times (3500–4000 million year ago) and probably helped precipitate and concentrate organic carbon-rich matter (Parnell, 2004). The first scientific recordings indicating that radioactivity affects living matter dates back to late 19th and early 20th century when Marie Skłodowska-Curie mentioned in her thesis that “The action of radium upon the skin can take place across metal screens, but with weakened effect” (Richards, 1915). Similarly, Henri Becquerel observed negative effects of radioactivity on his own body, after he carried a small tube of impure radium in his pocket for a few hours (Baskerville, 1905). Early experiments on the effect of ionizing radiation (IR) on plants were performed during late 19th and early 20th century (Gager, 1908). It was soon realized that radiation is a powerful mutagen (Nadson and Philippov, 1925), can induce variations within species (Goodspeed and Olson, 1928; Olson and Gilbert, 1928), and can control rates of mutations (Babcock and Collins, 1929).

Plants can easily cope with increased levels of IR. This has been demonstrated in the radio-contaminated Chernobyl (Shkvarnikov, 1990) and Fukushima (Mimura et al., 2014) environments, as well as their successful growth in space (Dubinin et al., 1973). Plant radio-resistance is maybe not surprising since radioactive materials occurred on the Earth’s surface when plants first appeared during the Mid-Ordovician period, about 460–470 million years ago (Wellman and Gray, 2000; Karam and Leslie, 2005). It has also been proposed that present day areas with high-levels of background natural radiation and the reduced levels of plant migration may have both contributed to plant radio-resistance (Moller and Mousseau, 2013). To investigate the molecular aspects of this process in plants, various analyses have been undertaken (Moller and Mousseau, 2015). Recent meta-analysis of 45 published studies on DNA mutations in Chernobyl showed that plant growth in radio-contaminated environment is associated with increased levels of mutation (Moller and Mousseau, 2015). It appears that DNA methylation and increased extra chromosomal homologous recombination events also contribute to successful plant growth in radio-contaminated environments (Kovalchuk et al., 2003, 2004).

However, transcript expression and protein abundance are found to poorly correlate (Chen et al., 2002; Griffin et al., 2002; Orntoft et al., 2002; Pascal et al., 2008; Hornshoj et al., 2009; Hajduch et al., 2010), including plants growing in radio-contaminated areas. Therefore, the complementation of expression studies with proteomics can provide new insight into molecular mechanisms of plant growth in radio-contaminated environments. Indeed, proteome alterations induced by IR are the subject of increased research interest, especially in mammalian systems (Azimzadeh et al., 2014; Leszczynski, 2014). In plants, this is also appears to be the case, as differential abundances of proteins associated with defense and stress responses were detected in leaves harvested from rice grown in the soil taken around Chernobyl reactor site (Rakwal et al., 2009). Importantly, it has been demonstrated that proteome changes increase with irradiation dose; observations were based on the analysis of X-rays irradiated plantlets of the reference plant *Arabidopsis thaliana* (Gicquel et al., 2011).

## Experimental Design of the Chernobyl Seed Project

**FIGURE 1:**
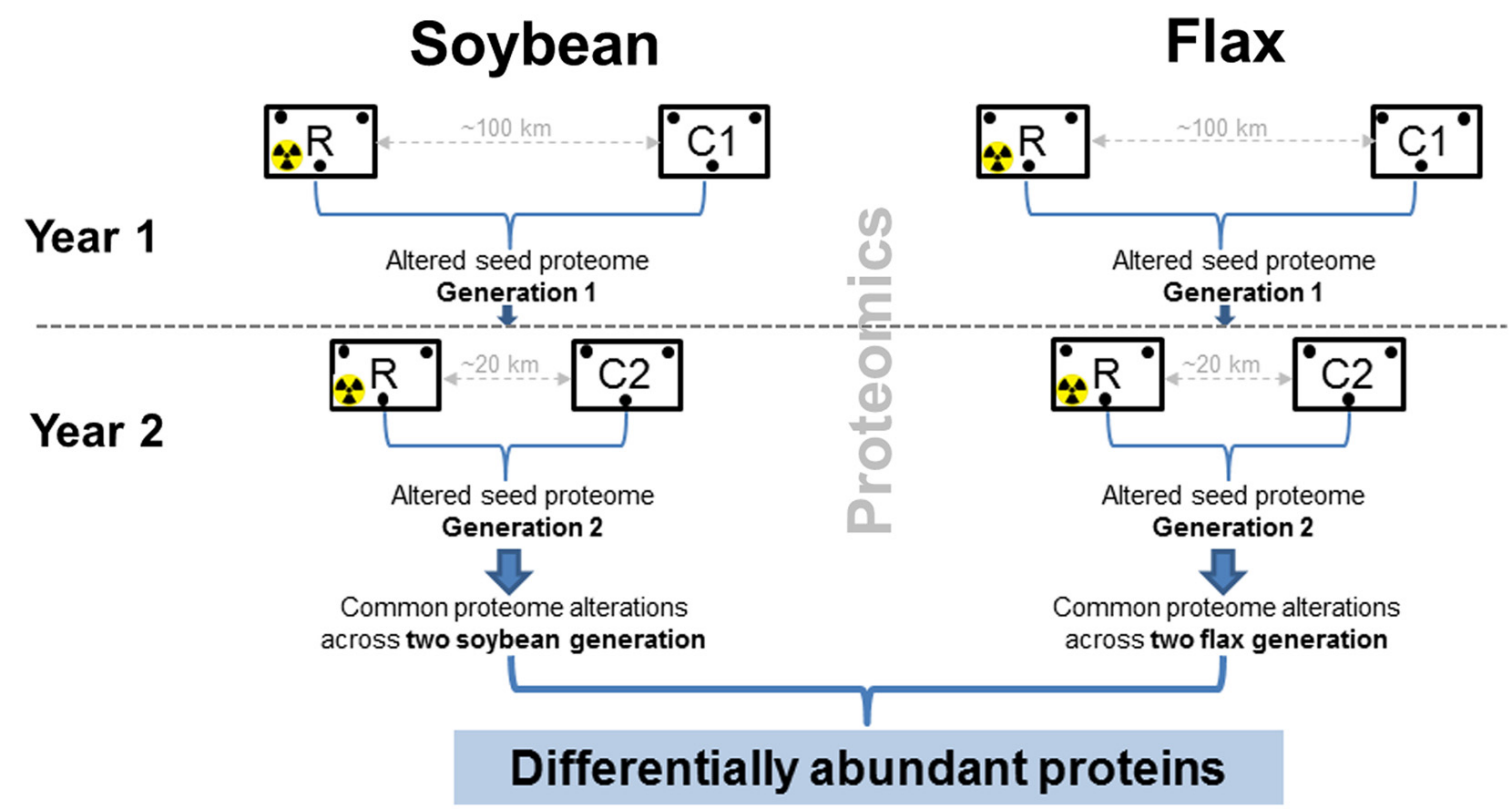
**Experimental design in the Chernobyl area during the two-year proteomic survey.** In the first year, local varieties of soybean (*Glycine max* [L.] Merr., variety Soniachna) and flax (*Linum usitatissimum*, L., variety Kyivskyi) were planted in radio-contaminated (R) and non-radioactive (C1) experimental fields in the Chernobyl area (**Supplementary Figure S1**). Seeds were harvested in biological triplicate and subjected to proteomic analyses. The following year, seeds not used for the analyses were planted into the same radio-contaminated field (R), but different non-radioactive (C2) experimental fields to obtain seeds from the second generation. To exclude alterations in seed proteomes related to field locations, only those differentially abundant proteins commonly observed across the two soybean and flax generations were considered.

Experimental design for ecological field experiments should include several experimental fields to avoid pseudoreplication (Hurlbert, 1984). However, it is often difficult to establish and manage several experimental fields in heavily controlled radio-contaminated areas. Therefore, experimental design for Chernobyl (**Figure 1**) was modified and included (i) two non-radioactive fields (control) and one radio-contaminated experimental field (**Supplementary Figures S1A,B**), (ii) two plant species, and (iii) two successive years. An important aspect of this experimental design (**Figure 1**) is the changed location of the non-radioactive field after the first year. The logic behind this is to exclude alterations related to the differences between experimental fields (soil, pests, weather, etc).

In 2007, local varieties of soybean (*Glycine max* [L.] Merr., variety Soniachna) and flax (*Linum usitatissimum*, L., variety Kyivskyi) were sown in radio-contaminated experimental fields (soil radioactivity 20650 ± 1050 Bq.kg^-1^ of ^137^Cs, and 5180 ± 550 Bq.kg^-1^ of ^90^Sr) located near the village Chistogalovka approximately 5 km from the Chernobyl Nuclear Power Plant (CNPP). The non-radioactive control experimental field (1350 ± 75 Bq.kg^-1^ for ^137^Cs and 490 ± 60 Bq.kg^-1^ for ^90^Sr) was established near Zhukin, a village approximately 100 km from CNPP (**Supplementary Figure S1A**). Soybean and flax seeds were harvested and mature seed proteomes comparatively analyzed in biological triplicate (**Figure 1**). In 2008, soybean and flax seeds harvested from the first generation of plants were sown onto the same radio-contaminated field, but a different non-radioactive field in the Chernobyl area (**Supplementary Figure S1B**), to obtain the second generation of seeds. A new non-radioactive experimental field was established directly in the town of Chernobyl, in an area with soil radioactivity of 1414 ± 71 Bq.kg^-1^ of ^137^Cs and 550 ± 55 Bq.kg^-1^ of ^90^Sr (**Supplementary Figure S1B**). The Chernobyl area is characterized by sod-podzolic soil (pH5.6–pH6.6, 12% clay, 2.0% organic compounds) which is a typical soil in the Ukrainian region of Polessia. Generally, in this area, the content of aleurite (silt) and pelitic soil ranges from 20 to 30% (Rashydov and Malinovskiy, 2002).

## Advances in the Establishment of Protein Abundance Profiles and Web-Based Database

In soybeans of the first generation, only 9.2% 2-DE spots, out of 698 quantified, were found differentially abundant between mature seeds harvested from non-radioactive and radio-contaminated Chernobyl areas (Danchenko et al., 2009). Similar to this, the analysis of the first generation of mature flax seeds showed differential abundance only in about 4.9% of resolved features from 720 quantified 2-DE spots (Klubicova et al., 2010). However, the results from these initial soybean and flax generations do not represent a large enough dataset upon which it is possible to base solid conclusions; it appears that growth in radio-contaminated environments has a relatively small effect on the seed proteome. Similar effects of IR have been previously shown on animal proteomes (Park et al., 2006; Guipaud et al., 2007) and support the notion that the exposure to low levels of IR do not significantly alter overall metabolism. Such speculation may be further supported by a study on the roots of the reference plant *Arabidopsis thaliana* under low levels of ^137^Cs, where only a small percentage of the transcriptome was differentially expressed (Sahr et al., 2005).

**FIGURE 2:**
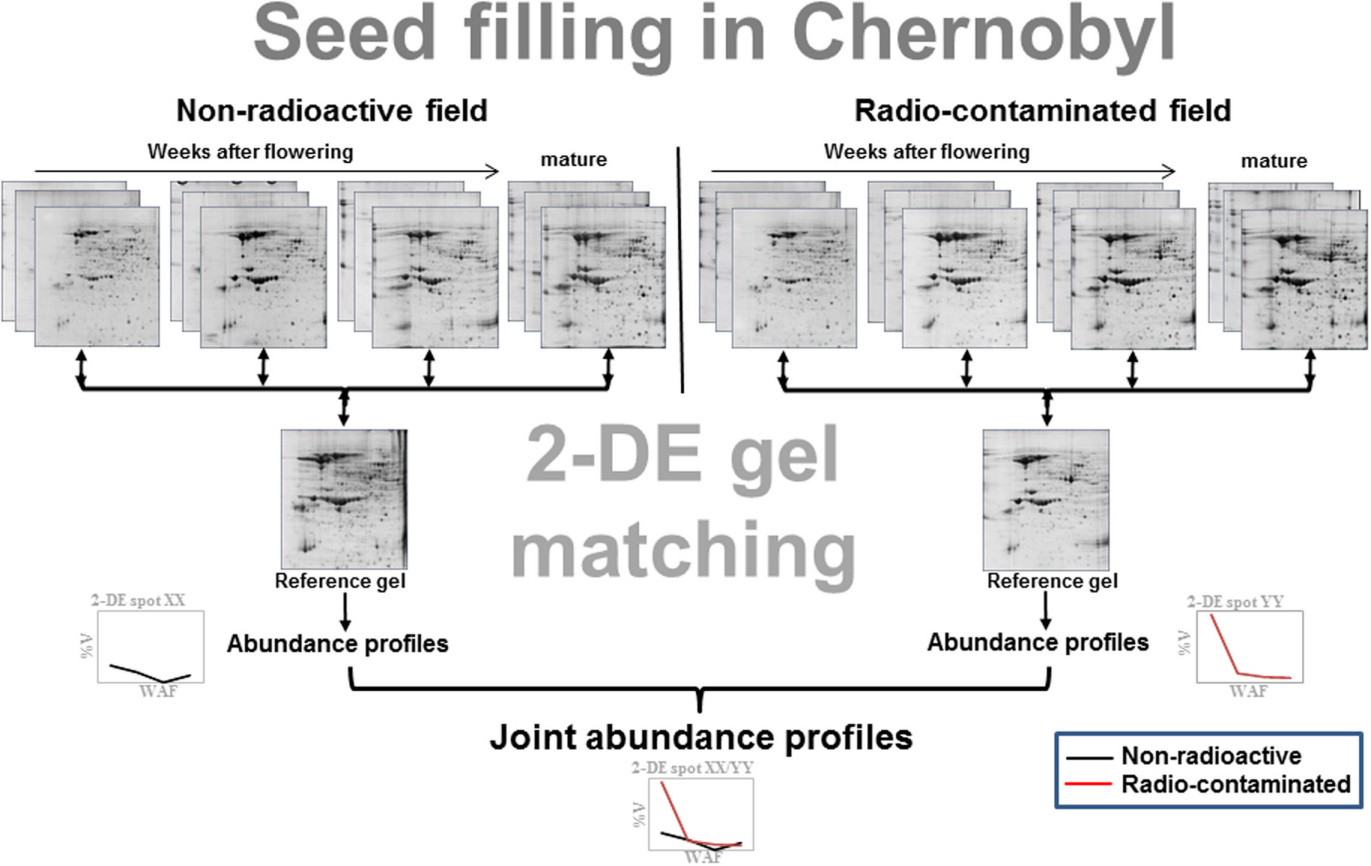
**Schematic overview on the establishment of protein abundance profiles – modified from Klubicova et al. (2012a)**. Briefly, developing soybean seeds were harvested at 4, 5, 6 weeks after flowering (WAF) (flax seeds at 2, 4, and 6 WAF) and at a mature stage from plants grown in non-radioactive and radio-contaminated Chernobyl experimental fields (**Supplementary Figure S1**). Isolated total protein was resolved by two-dimensional protein electrophoresis (2-DE) in biological triplicate. The 2-DE gels were matched to the pooled (reference) gels using ImageMaster 4.9 software. Finally, abundance profiles from both experimental fields were matched and joint abundance profiles, i.e., profiles for the same spot across seed filling in non-radioactive and radio-contaminated experimental fields, were established.

In order to provide a more detailed insight into the seed proteome in radio-contaminated environments, protein abundances profiles were established from developing soybean and flax seeds (**Figure 2**) from the second generation which were harvested from both Chernobyl experimental fields (**Supplementary Figure S1B**). Protein abundance profiles are capable of comprehensively characterizing protein abundances during seed filling. The approach has been used successfully in soybean (Hajduch et al., 2005), canola (Hajduch et al., 2006), castor (Houston et al., 2009), and *Arabidopsis* (Hajduch et al., 2010). In these Chernobyl studies, protein abundance profiles were first established for each experimental field and then matched to obtain joint abundance profiles (**Figure 2**). Using this approach, it was possible to provide a detailed overview of protein abundances during seed filling in soybean (Klubicova et al., 2012a) and flax (Klubicova et al., 2010) across both experimental fields. For instance, it was revealed that β-conclycinin significantly decreased during seed filling in radio-contaminated areas in the second soybean generation (Klubicova et al., 2012a). These analyses also revealed alterations of proteins associated with carbon metabolism in the cytoplasm and plastids and to the carboxylic acid cycle in the mitochondria (Klubicova et al., 2012a). In flax, increased abundance of proteins associated with isocitrate dehydrogenation, L-malate decarboxylation, pyruvate biosynthesis, and ethanol oxidation to acetaldehyde were detected at early stages of seed filling (Klubicova et al., 2013).

The data from these experiments can be viewed in a user-friendly format at dedicated web-based database that is freely available at www.chernobylproteomics.sav.sk. The aim of this online data depository is to allow scientific community (but also general public) to access the data from this project in user-friendly format. At the time of the database establishment (Klubicova et al., 2012b) the database contained the data from first, second soybean and first flax generation. Since then, the data from second flax (Klubicova et al., 2013) and third (Gabrisova et al., in review) generations were uploaded.

## Chernobyl Seed Project Suggested the Identity of Proteins Putatively Associated with Plant Growth in Radio-Contaminated Environments

The aim of these studies was to detect alterations in seed proteomes related to the radio-contaminated environment. However, the alterations in seed proteomes described above might also be associated with the differences between the experimental fields (soil, pests, weather etc). To exclude this possibility, data were further analyzed and alterations common for both plant generations and plant species identified.

Altered abundance of enzymes associated with the glycine betaine biosynthetic pathway was jointly detected in the first generation of soybean (Danchenko et al., 2009) and flax (Klubicova et al., 2010). It is tempting to speculate that glycine betaine is involved at early stages of plant response toward the radio-contaminated environment. Interestingly, the involvement of glycine betaine in protection against IR was shown previously in human blood (Monobe et al., 2005). Since plants with altered levels of glycine betaine have already been produced (Waditee et al., 2007) it should be possible to directly test the putative protective role of glycine betaine in radio-contaminated environments.

The mobilization of seed storage proteins (SSP) and alteration of proteins associated with carbon assimilation and fatty acid metabolism were observed jointly in both generations of soybean and flax. These data support the notion that SSPs are involved in seed defense against various threats, as has been shown previously with their role in defense against Bruchids (Sales et al., 2000). Interestingly, the application of salicylic acid during germination *Arabidopsis thaliana* resulted in mobilization of SSPs (Rajjou et al., 2006). Furthermore, it has been proposed that class 2S albumin SSPs are defensive proteins (Regente and De La Canal, 2001), while salt stress has been shown to alter the abundance of β-conglycinin SSP (Aghaei et al., 2009).

An interesting aspect of these Chernobyl studies are differential abundance of proteins associated with carbon assimilation and fatty acid metabolism in both generations of soybean and flax. As a result of this, the second generation of soybean (Klubicova et al., 2012a) and flax (Klubicova et al., 2013) showed altered total oil content in mature seeds. However, additional studies are needed to determine whether altered seed oil content is the result of genetic mutation or has an epigenetic or posttranslational explanation.

## Studies in Fukushima Radio-Contaminated Environment

Similar to the disaster at the CNPP in 1986, the accident at the Fukushima Daiichi Nuclear Power Plant in 2011 contaminated large areas with radioactivity (Buesseler et al., 2011; Kinoshita et al., 2011; Yasunari et al., 2011). Unfortunately, nuclear accidents provide unexpected justifications for research aimed at understanding plant survival and adaptation in radio-contaminated environments. Indeed, Hayashi at al. (2014) performed a pioneering study in the Fukushima radio-contaminated areas through the investigation of rice seedlings under continuous low-dose radiation. This study provided an overview of the transcriptome response in rice toward low level of gamma radiation and identified large numbers of genes with altered expression patterns (Hayashi et al., 2014).

It will be interesting to compare results from the Chernobyl studies using similar experimental setups in the Fukushima radio-contaminated area. The web based database (chernobylproteomics.sav.sk) might be a good tool for quick data comparison. Ideally, follow-up studies in Fukushima should include several non-radioactive and radio-contaminated experimental fields to avoid pseudoreplication (Hurlbert, 1984). If this is not feasible due to restricted/closed areas, an experimental design for radio-contaminated areas presented in this current study (**Figure 1**) could be applied.

## Conclusion

The outcome of these Chernobyl studies was the identification of several proteins with differentially abundances in soybean and flax seeds harvested during two successive generations. It is tempting to speculate that these proteins are associated with plant growth and adaptation in radio-contaminated environments. However, follow-up studies in both the Chernobyl and Fukushima radio-contaminated areas are required to further develop these hypotheses.

## Conflict of Interest Statement

The authors declare that the research was conducted in the absence of any commercial or financial relationships that could be construed as a potential conflict of interest.

## Supplementary Material

Figure S1 **The location of the Chernobyl experimental fields**. The radio-contaminated field (R) was established 5 km from Chernobyl Nuclear Power Plant (C), within the exclusion zone (D). The non-radioactive field (C1)was initially established in 2007 about 100 km from C **(A)** but since 2008 the non-radioactive field (C2) has been transferred directly to the town of Chernobyl **(B)**.
