# Supplementary figures and images for: Chernobyl seed project. Advances in the identification of differentially abundant proteins in a radio-contaminated environment
Source: Front Plant Sci. 2015 Jul 6;6:493. doi: 10.3389/fpls.2015.00493 (PMC4492160; doi:10.3389/fpls.2015.00493)

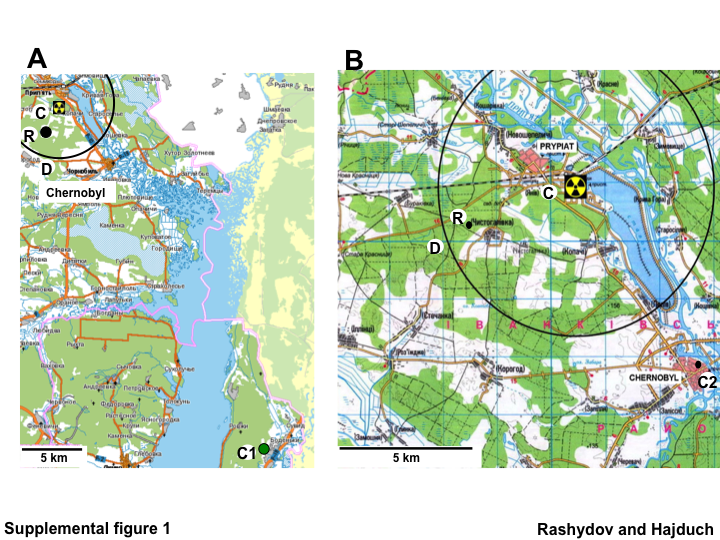

Supplement: Figure S1 — The location of the Chernobyl experimental fields. The radio-contaminated field (R) was established 5 km from Chernobyl Nuclear Power Plant (C), within the exclusion zone (D). The non-radioactive field (C1)was initially established in 2007 about 100 km from C (A) but since 2008 the non-radioactive field (C2) has been transferred directly to the town of Chernobyl (B). [file Supplemetary_Figure_S1.TIFF]
